# Helping Québec Pharmacists Seize the Vaccination Service Opportunity: The Pharmacy Best Practice Workshops

**DOI:** 10.3390/pharmacy9010051

**Published:** 2021-03-03

**Authors:** Kajan Srirangan, Arnaud Lavenue

**Affiliations:** Toc Toc Communications, 104-7030 Rue Marconi, Montréal, QC H2S 3K1, Canada

**Keywords:** pharmacists, vaccines, vaccine hesitancy, immunization programs, education, workshop training

## Abstract

Vaccinations are a safe and effective way to protect against infectious diseases. The World Health Organization estimates vaccines have saved more lives than any other interventions and every year about two to three million deaths are averted worldwide through immunization. To improve vaccination coverage, pharmacists have been increasingly involved in immunization roles in their communities—as advocates, educators, and immunizers. Community pharmacy-based vaccination services have increased both in the number of immunization providers and the number of sites where patients can receive immunizations. In Canada, health care is under provincial legislation—and so, there are distinct differences in scope of pharmacist practice across the country. Prior to the COVID-19 outbreak in early 2020, in Québec, Canada’s second-largest province, pharmacists did not have the authority to administer vaccines. To help prepare pharmacists in Québec to become immunizers, we developed and deployed a series of accredited workshops. In these facilitated workshops, pharmacists were able to share best practices that may lead to providing effective vaccination services, identify common competency gaps, discuss effective patient communication skills, and determine how to target the most vulnerable population groups. Participants were also asked to evaluate the workshop. Our results indicate the evaluation was very reliable in measuring participant satisfaction (Cronbach’s α = 0.94) and pharmacists commented that the workshops’ learning outcomes exceeded their expectations, and the topics covered were relevant and applicable. The evaluation also asked participants to identify weaknesses of training, so future educational interventions can be planned accordingly. We believe this work will contribute to the continual growth and advancement of the pharmacy profession in Canada.

## 1. Introduction

Over the last 50 years, immunization has saved more lives in Canada than any other single health care intervention [1]. Despite efforts by health authorities to encourage more vaccinations, suboptimal uptake of vaccines remains both an ongoing Canadian and global issue [2,3,4,5,6]. In addition to pediatric immunization efforts, the Canadian Immunization Guide (CIG) advises that prevention of infectious diseases by immunization should be pursued throughout life, and that all adults should be appropriately vaccinated [7,8]. Reasons for adult vaccinations include booster doses, updates to current vaccinations, changes in sexual risk behaviour, risk of professional exposure, and travel and immigration [8,9]. Some vaccinations, recommended specifically for adults, are also fairly new, such as vaccines for human papillomavirus (HPV) and shingles (herpes zoster) [10,11]. Nevertheless, despite the benefits of immunization, skepticism about vaccinations and general inconvenience continue to deter people from getting vaccinated [12]. Additionally, the issue of “vaccine hesitancy” (i.e., delay in acceptance or refusal of vaccines despite their availability) is a well-recognized problem in Canada and globally [13]. As noted by Macdonald (2016) [7], it has become increasingly difficult to ensure timely acceptance of vaccines by provincial and territorial immunization programs or front-line health care professionals. Furthermore, providing more public information on the benefits of vaccines is not enough, and overselling by emphasizing the facts about the benefits and disease risks ostensibly leads to further hesitancy and suboptimal vaccine uptake [14,15]. The Government of Canada’s adult National Immunization Coverage Survey (aNICS) in 2016 revealed that less than half of the adults (~40%) received a dose of the influenza vaccine for the 2015–2016 season [16]. As reported by the Canadian National Advisory Committee on Immunization (NACI), influenza, along with pneumonia, ranks among the top 10 leading causes of death in Canada [17,18]. While the disease burden of influenza can vary from season to season, it is estimated that there is an average of approximately 12,200 hospitalizations [19] and 3,500 deaths [20] related to influenza per year. In addition to influenza and pneumococcal vaccinations, equally concerning are the low vaccination rates for other adult vaccinations [21]. For instance, the 2016 aNICS [16] found that the national pertussis vaccination coverage was low, as ~10% of adults reported having received a booster dose of the pertussis vaccine in adulthood. The survey also found that 54% of adults reported having been vaccinated against tetanus within the last 10 years. Likewise, NACI recommends that adults 50 years of age and older receive one dose of shingles (herpes zoster) vaccine [22]. According to the 2016 aNICS, among individuals 50 years of age and older, ~20% reported having received the shingles (herpes zoster) vaccine [16]. In an effort to increase vaccination rates in adolescents and adults, all provincial governments in Canada have expanded the scope of practice of pharmacists to include the administration of influenza and other critical vaccines. This expanded scope of practice has also been implemented in Yukon, but not in the other territories (i.e., Northwest Territories and Nunavut) [23,24]. In Canada, the support for broadening of the scope of pharmacists’ practice and nurses began in 2003, based on a growing recognition that patients’ health care demands are exceeding physicians’ capacity to address them. The need for reforms to patient care and experience and the role of allied health professionals was put forth by the Canadian Medical Association, the Canadian Pharmacists Association, and the Canadian Nurses Association [25,26]. The statement by these associations reinforced the view that policy initiatives needed to be taken at national, provincial, and territorial levels to expand the scope of practice of health professionals in order to improve support for patients and give them greater access to resources [25].

In addition to involvement in immunization programs, hospital- and community-based pharmacists in select Canadian jurisdictions can renew, refuse to fill, adjust and/or substitute prescriptions, and order laboratory tests. Given that pharmacists may be the first members of the health care team to recognize patients’ needs, they are becoming increasingly involved in patient education, providing access to medications, chronic disease management services, tobacco cessation interventions, and cardiovascular screening. In front-line care, pharmacists are uniquely suited to tackle these additional roles, given their educational training, but are all too often underutilized due to lack of communication and collaboration with other allied health professionals, shortage of shared vision about services provided by pharmacists, and lack of remuneration for expanded services [27]. Internationally, pharmacists are also increasingly becoming involved in joint patient care. Countries such as the United States, United Kingdom, and New Zealand have all moved toward various forms of independent or collaborative prescribing [28,29,30,31]. While the type of vaccine administered and/or prescribed by pharmacists varies by country, it generally includes vaccines for influenza, pneumococcal, HPV, as well as travel-related vaccines [32]. To develop competency to administer vaccinations, pharmacists undergo training either as part of an undergraduate pharmacy qualification or through supplemental pharmacist-specific vaccination training programs available after undergraduate studies. For instance, in most Canadian provinces and territories, where pharmacists have injection authority, competency expectations have been standardized by the National Association of Pharmacy Regulatory Authorities (NAPRA). Therefore, training programs approved by each jurisdiction are quite similar, and are accredited by the Canadian Council on Continuing Education in Pharmacy (CCCEP) or l’Ordre des pharmaciens du Québec (OPQ). Internationally, and in Canada, there is robust evidence from studies that pharmacist involvement in vaccine delivery improves outcomes and patient access, increases vaccine uptake, decreases disease burden, and generally leads to an overall positive economic and social impact [33,34,35,36,37,38]. For example, a recent study surveyed 1,502 adults, who received pharmacist-administered seasonal flu vaccination at four different community pharmacy locations in Toronto, Ontario, Canada, about their perceptions toward pharmacists as immunizers (PAIs) [39]. In addition to reporting a high degree of patient satisfaction (~91.8% of the patients were very satisfied with the pharmacist’s services and injection technique), the study pointed out that ~25% of the patients surveyed were not regular annual vaccine recipients and ~25% were identified as high-risk according to previously published criteria by the Public Health Agency of Canada. More importantly, ~28% of the total patients and ~21% of the high-risk patients would not have received seasonal flu vaccinations that year if it were not available via a pharmacy.

While all Canadian provincial jurisdictions have granted pharmacists the authority to vaccinate; Québec was the last province to do so. From June 2019 to March 2020, the government of Québec only tabled a provincial legislation (i.e., Bill 31) to extend vaccination authority to pharmacists [23,24]. Prior to March 2020, pharmacies in Québec were therefore largely limited to distributing vaccines. However, during this time, Québec pharmacists still contributed substantially to vaccination efforts by promoting vaccinations, providing counselling, and hosting onsite nurses to administer the vaccines [23]. In March 2020, in light of the COVID-19 pandemic, the government of Québec accelerated the passing of Bill 31, granting Québec pharmacists the ability to prescribe and administer vaccines. In anticipation of the changes to their scope of practice, some pharmacists wanted to be better equipped with counselling and management skills of vaccine-preventable diseases (VPD). Equally importantly, pharmacy owners also struggled to integrate vaccination services or did not fully understand the value of pharmacists’ involvement in the delivery of vaccination services. To address these issues, we developed and deployed facilitated interactive workshops on pharmacy-based vaccination practices specifically targeting pharmacy owners in Québec, with the aim of making vaccination services (e.g., identifying and managing patients, making recommendations, and integrating vaccination services into the day-to-day operations) easy to implement. Our workshops also focused on elevating the collaborative work between the pharmacists and other allied health care workers, such as pharmacy nurses.

## 2. Methods

### 2.1. Identification of Best Practices and Competency Gaps

To develop the interactive workshop, we first sought to determine the best practices, opportunities, ambiguities, and barriers that currently persist in the province of Québec for pharmacies undertaking vaccination services. To critically explore this, field work was undertaken to identify pharmacy owners/practices that were working collaboratively and proactively with nurse immunizers to provide vaccination services. Of note, we specifically wanted to observe pharmacy services that were patient-centric and provided adult vaccination services year around (i.e., well beyond influenza vaccines that are administered during scheduled vaccination days in the province). Identification of community pharmacies exhibiting these qualities was done, in part, through canvassing different locations in Montréal, Québec. Furthermore, to frame an effective response to these issues, we additionally sought the assistance from pharmacy owners and pharmacists in community pharmacies. Building upon their knowledge, we were able to ascertain which pharmacies were more proactive in providing vaccination services than others. Snowballing techniques were also used by asking participating pharmacists to refer other community pharmacists that were exhibiting similar qualities of providing effective vaccination services. A period of six months (totalling approximately 100 h) was spent observing, interacting with, and interviewing pharmacy owners and nurse immunizers in the community setting. The findings from the field survey were then collated, processed, and analyzed by a pharmacist to identify the common themes and overarching issues present in the province. These themes were further streamlined into coherent narratives to be disseminated as talking points during the workshop.

### 2.2. Development of Clinical Studies

Another important facet to the training was to ensure that pharmacists feel equipped with counselling and management skills of vaccine-preventable diseases. Equally importantly, pharmacists should be able to identify and manage the vulnerable population groups that can be recommended for vaccinations. To tackle these fronts, with the assistance of a registered and credentialed pharmacist of a large community pharmacy, a series of scenario-based patient profiles (i.e., patients’ characteristics relevant to VPD-risk) was developed.

### 2.3. Developing the Expert-Led Interactive Workshops

**Figure 1:**
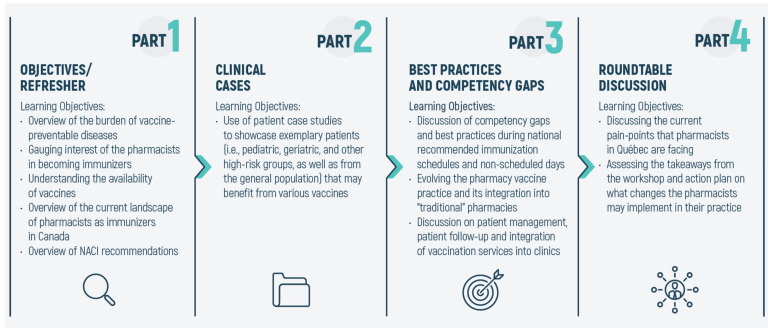
Workshop structure and content. The structure of the workshop was organized into four segments: (**1**) a refresher providing an overview of vaccine-preventable disease and National Advisory Committee on Immunization (NACI) guidelines, (**2**) a set of clinical cases showcasing exemplary patients that may benefit from vaccinations, (**3**) an overview of our fieldwork and findings on effectively implementing pharmacy-based immunization clinic and common competency gaps to avoid, and (**4**) a roundtable discussion with pharmacy owners on the evolution of their profession.

As shown in Figure 1, these two components (i.e., the clinical cases and the best practices/competency gaps) formed the cores of the workshop. The workshop was further fortified to include a refresher/introduction to the workshop and a final roundtable discussion. The refresher focused on laying the groundwork for the workshop and providing the pharmacists with an up-to-date landscape of vaccination authority across Canada. In the introduction portion, we also discuss the current NACI recommendations for vaccines and the current vaccinations available to the adult population in Canada. The opening session of the workshop was also used to investigate and gauge the pharmacists’ interest in becoming PAIs in Québec. Finally, the fourth and last portion of the workshop was the roundtable discussion. The roundtable discussion served two major purposes: First, in line with current developments in this field, it opened the floor for a more outward-looking approach on PAIs and what the participants felt were the current challenges. Second, the pharmacists were asked to assess the learning outcomes of the workshop (see section below) and whether they were relevant and applicable to their practice. The evaluation also asked participants to identify weaknesses or deficits of the training module, so that future educational interventions can be planned accordingly.

### 2.4. Evaluation of the Workshop

Upon completion of the workshop on vaccination, pharmacists were asked to complete an anonymous evaluation questionnaire. The evaluation questionnaire covered all major facets of the training workshop, and its completion was mandatory for a continuing education unit credit from OPQ. As all participants were French-speaking, the evaluation questionnaire was written and completed in French. The English translation of the questionnaire is available as Supplementary Material. The evaluation questionnaire used a 5-point Likert scale for the first five parts, consisting of 30 items, with anchors “low” to “very high”. Part 1 of the evaluation focused on professional development and asked pharmacists whether the workshop improved their knowledge and skills of vaccinations, and whether they would recommend the workshop to colleagues. Part 2 directly focused on evaluating the workshop’s learning outcomes and whether it was serving the goals that were established. Part 3, on the other hand, evaluated the clinical cases that were presented and whether they were relevant, sufficient, applicable, and diversified. Part 4 evaluated the workshop facilitator’s knowledge of the subject, and their ability in fostering interactivity, collaboration and cooperative learning, and whether they motivated effective group participation. Part 5 focused on whether the workshop was suitable as a certification from OPQ. Lastly, in the free-text portion of the evaluation, pharmacists were able to provide qualitative assessments on the strengths and limitations of the workshop. They were also asked to identify potential topics to be covered in future educational workshops. Descriptive statistics were used to summarize and describe the evaluations, and the Cronbach α coefficient was used to verify internal consistency. Statistical analysis was performed using SPSS version 20 software (SPSS Inc., Chicago, IL, USA). Free-text responses were analyzed using a thematic approach and word frequency was calculated using Frontline Systems’ Solver 2019 (Frontline Systems Inc., Incline Village, NV, USA) add-in for Microsoft Office Excel 16.0 (Microsoft Corporation, Redmond, WA, USA).

## 3. Results and Discussion

**Figure 2:**
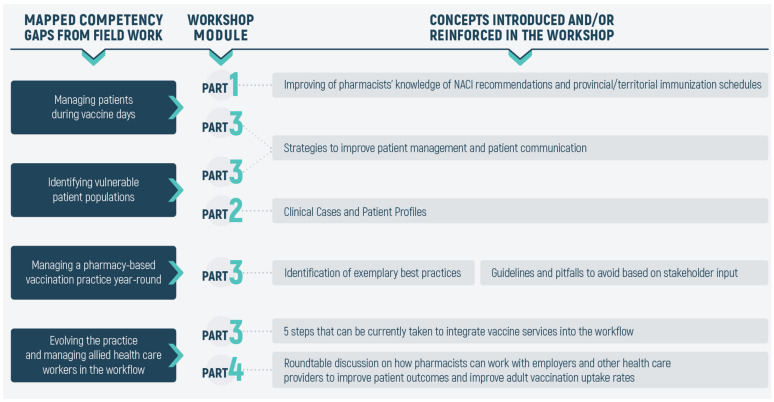
Identified competency gaps and their introduction into the workshop training modules. From our fieldwork, it was apparent that pharmacists had four major competency gaps: (**1**) effectively managing patients during scheduled national and provincial vaccine days, (**2**) identifying vulnerable patient populations, (**3**) year-round management of a pharmacy-based vaccination clinic, and (**4**) managing allied health care workers (i.e., immunization nurses, pharmacy technicians, etc.) and integrating them into the workflow. Various strategies and solutions to close these competency gaps were identified and introduced into the workshop training modules.

From our field work, it was observed that pharmacists generally felt well-prepared to become immunizers and possessed similar levels of competency to that of nurse immunizers. However, the following gaps were observed: (1) managing patients during scheduled vaccine days, (2) identifying vulnerable populations and providing them with evidence-based advice about vaccinations, (3) effectively managing a pharmacy-based vaccination practice, and, finally, (4) identifying strategies to evolve the practice further (as laws change), and how to manage allied health care workers (e.g., nurse immunizers) in the workflow. As shown in Figure 2, these concepts were therefore incorporated into one or more of the four major parts of the workshop. The workshop began with a panel discussion to gauge the interest of pharmacists becoming immunizers in Québec. This was followed by an overview of the laws and regulations regarding PAI, at the provincial and territorial levels. Next, to address the first competency (i.e., improving the management of patients during scheduled vaccination days), we discussed the overall vaccination rates among adults in Canada, and delved into assessing the pharmacist’s awareness of the NACI recommendations and their knowledge about important adult vaccines. Additionally, an overview of the mechanism of action of vaccines and adjuvants was presented. It was our hope that this section would allow the participants to gain a perspective to how low vaccine coverage influenced disease occurrence among Canadians, which would ultimately be communicated to patients.

The next major section (i.e., Part 2) of the workshop was designed to fulfill the competency gaps that the pharmacists may have regarding the identification and management of vulnerable populations where vaccinations carry heightened importance. In using clinical cases and various patient profiles, the major learning objectives here were to ensure that pharmacists felt equipped with necessary knowledge, attitudes, and preparedness to promote and educate vaccines for vulnerable populations. As mentioned, in using invasive pneumococcal disease as a reference condition, we developed a variety of different patient profiles with underlying pathologies or conditions that may benefit from pneumococcal vaccination.

**Figure 3:**
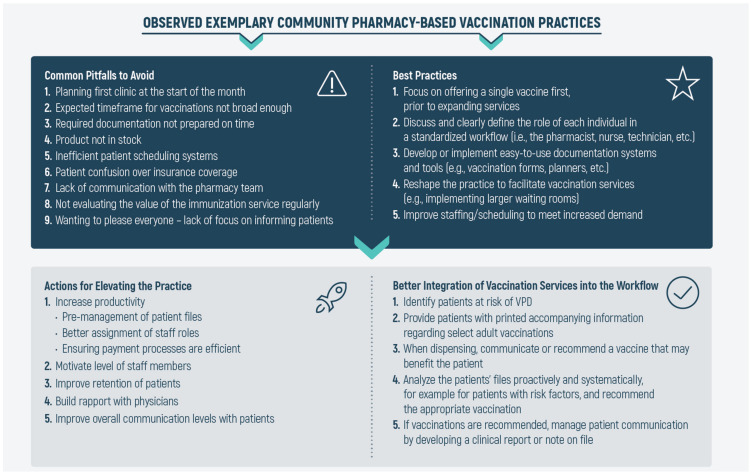
Observed exemplary best practices from the community. From our fieldwork, we observed common pitfalls that a pharmacy-based vaccination practice should avoid and the best practices from exemplary clinics. Based on these observations and on data obtained from the literature, we propose the following ways to better integrate vaccination services into a pharmacy-based vaccination clinic and improve existing clinics to respond to the needs and barriers of patients more adequately.

The competency gaps were addressed in Parts 3 and 4 of the workshops and involved tackling issues related to managing a practice that provides full-fledged vaccination services and how to evolve the practice in light of changing regulations. As summarized in Figure 3, the first competency gap was addressed via our findings from our field work, in which we mapped common pitfalls that a pharmacy-based vaccination practice should avoid and the observed best practices from exemplary clinics. Common pitfalls to avoid included: (1) inadequate planning of clinic days for routine immunization schedule, (2) expected timeframes for the vaccinations offered are not broad enough, (3) ill preparation of documentation, which in turn delays the overall schedule, (4) not carrying stock of vaccines (particularly travel vaccines), needles, and syringes, (5) inefficient patient scheduling systems, (6) patients becoming confused over coverage of vaccinations and injection charges, (7) lack of communication with the pharmacy team over their roles, objectives, and overall goals, (8) not evaluating the value of the immunization service regularly, and finally, and (9) lack of focus on informing patients about the value of vaccination and the services offered by the clinic. Similarly, in examining clinics that support vaccination services and in conducting semi-structured interviews with pharmacists, the developed best practices framework included: (1) in initiating vaccination services, it was best to focus on one vaccination service prior to expanding the service offering, (2) holding team meetings to explain which services are provided by the clinic, and the role of each member of the team (pharmacist, technician, cashier, etc.), (3) developing easy-to-use tools and documentation (e.g., appointment planners and vaccination forms), (4) depending on the volume of patients expected, it was often best to reshape the practice and implement larger waiting rooms, and (5) improving staffing and scheduling in order to meet the increased demand. As further illustrated in Figure 3, by enforcing these best practices and avoiding pitfalls, pharmacy owners can effectively start to evolve their practice and incorporate vaccination services into the regular workflow.

A total of 57 pharmacists across the province completed the initial workshop and received continuing education unit credit from OPQ. As mentioned in Section 2, to receive an accreditation credit by OPQ, completion of the evaluation questionnaire was mandatory. The questionnaire was reliable in measuring participant satisfaction across all aspects of the training workshop (Cronbach’s α coefficient = 0.924). The feedback from participants, as acquired from the questionnaire is summarized in Supplementary Figure S1. Overall, the participants were highly satisfied with the program, as indicated by the frequent median responses of 4 on a Likert scale of 1–5 for Questions 1 to 30 (overall mean: 4.69/5). Of note, in terms of professional development (i.e., Questions 1–3), participants recognized the value of the training activities and believed they would directly impact their practice. Participants gave the professional development section a mean rating of 4.63. Feedback from the objectives and content portion (i.e., Questions 4–8) was also overwhelmingly positive. The participants found that the learning objectives were clear, relevant, and complete, and gave the section a mean rating of 4.57. Equally importantly, participants also highly felt that the clinical cases (i.e., Questions 9–13) were a critical component of the workshop and that the clinical cases and scenarios were applicable, relevant, and diverse and gave the section a mean rating of 4.70.

Results from the questionnaire’s open-ended questions were collated and a thematic analysis was undertaken. Regarding the strengths of the workshop, most participants felt that the workshop was “practical”, “clear”, and “informative” and relayed positive comments regarding the workshop’s overall facilitation and its interactivity. Participants, however, felt the workshop could be improved by: (1) having more in-depth discussions about other adult vaccines and (2) increasing the time length of the workshop (i.e., from 2 h) to accommodate more clinical cases. Furthermore, as we envisioned this training workshop to be one in a series that would be offered to pharmacists in light of their expanded scope of practice, we also asked them to list potential themes they would want to see explored in future sessions. Participants identified six learning interventions that can be explored: (1) discussions on the role pharmacists can play in chronic obstructive pulmonary disease (COPD) and asthma management, (2) training on vaccine administration and discussion of other types of vaccinations (e.g., travel health, HPV, and meningitis), (3) discussion on pharmacy-based nurse services and the training for pharmacy-based nurses, (4) discussion on pharmacists’ involvement in therapeutic drug monitoring (TDM), (5) discussions on clinical follow-ups and collective prescriptions, and (6) role of pharmacists in caring for patients with HIV/AIDS. Six months after the workshop, we followed up with participants in a semi-structured interview to explore the utility of the workshop and whether it had an impact on their practice. Although only seven participants (~10.7%) from the original cohort took up this opportunity, almost all reported that the workshop was crucial in improving: (1) meetings with staff pharmacists and technicians, (2) organization of work with the nurses, and (3) targeting the at-risk population and handing out resources (e.g., pamphlets). However, many respondents indicated that major obstacles with the practice still include shortage of staff, mobilization of the team about immunization, and lack of time to plan for scheduled national vaccination days. Furthermore, respondents noted that future learning sessions could include modules focused on developing a personalized action plan for patients, development of communication plans for teams, and discussion of important clinical cases.

Other studies have also documented the impact of pharmacist-specific vaccination training programs designed to “retro-fit” the profession. Of note was the work by Lau et al. [40] on the development, implementation, and evaluation of the Queensland Pharmacist Immunisation Pilot (QPIP) workshops. The Pharmaceutical Society of Australia’s QPIP program worked with over 200 pharmacies to deliver training workshops to 339 participants. These workshops were initially implemented for the 2014 influenza season but were later expanded to encompass other vaccines (i.e., measles, mumps, and rubella and pertussis, diphtheria, and tetanus). In a similar spirit to our method, the QPIP workshops also mapped competencies for pharmacists against those of nurse immunizers to identify competency gaps in domains such as managing vaccines, managing deteriorating patients, and cold chain considerations. While our work focused on peer-assisted learning to manage vaccination clinics, the core component of the QPIP workshops was face-to-face practical training in injection skills and anaphylaxis management. Similar to our findings, participants of the QPIP workshops were also satisfied with the training outcomes, including the online pre-reading material and the hands-on training opportunity to practise injections on each other. In Western Australia, Hattingh and colleagues [41] evaluated, among other things, the challenges experienced by pharmacy staff in the preparation, implementation, and delivery of influenza vaccination services. Challenges experienced by the pharmacy-based vaccination clinics included staffing issues, maintaining stock, promotion of vaccination services and communicating with consumers. Findings such as this highlight the need for educational interventions aimed at improving the operationalization of pharmacy-based vaccination clinics. A fundamental aim of our work was to equip pharmacists with increased knowledge of critical adult vaccinations, including but not limited to influenza. More recently, the impact of targeted training programs for pharmacies participating in the provision of a variety of immunizations, beyond influenza vaccines have also been reported. For instance, Westrick et al. [42] evaluated the impact of the “R_x_Vaccinate” program for pharmacy-based pneumococcal immunization, where one group of pharmacists received only the self-directed training and the other group received both self-directed training and peer-coaching sessions. The authors observed significant increases in the number of pharmacist-administered pneumococcal vaccinations in both groups, however, the increase was greater in the group receiving both self-directed training and expert and peer coaching than the group receiving only the self-directed training. These results further exemplify the importance of peer-based learning processes and its influence on learning outcomes. Percy et al. [43] reported on the outcomes of a training program aimed at pharmacist-extenders (i.e., pharmacy technicians and interns) to improve vaccination rates within a community chain pharmacy. Like our work, their training program included patient cases (pneumococcal and herpes zoster (shingles) vaccine eligibility) and strategies for patient counselling and recommending vaccines. Overall, their findings highlight the crucial role patient cases play in training programs to help pharmacy and pharmacy staff identify eligible patients and improve vaccination rates. Finally, Merks et al. [44] reported on the readiness and willingness of pharmacists in Poland to provide immunization services after completing the “Pharmacists Without Borders” educational workshops during the COVID-19 pandemic (February 2020–August 2020). Relevant to our work, the authors indicated that part of the apprehension pharmacists have toward providing vaccination services seem to stem from increased workload, not enough vaccination training courses and pharmacies not being adjusted to provide these types of services.

## 4. Conclusions

Globally, professional retooling and legislative changes are taking place to expand the scope of pharmacists as vaccinators against influenza and other major vaccine-preventable diseases [45,46]. “The Pharmacy Best Practice Workshops” has demonstrated its effectiveness in equipping Québec pharmacists with the knowledge, skills and dispositions required to integrate vaccination services into the day-to-day operations, identify at-risk patients, and counsel and recommend vaccinations. Given the encouraging responses from the participants and the overall success of these workshops, we are now in the process of developing a second set of workshop modules. These newer modules will take into account the recommendations suggested by the participants of this study whilst delving deeper into topics such as improving the pharmacists’ competencies to prescribe and administer injections, further considerations required to integrate and optimize the roles of other allied health care workers (e.g., pharmacy nurses and technicians) into the practice, and the communication processes that are necessary to ensure optimal workflow integration.

## Supplementary Materials

The following are available online at https://www.mdpi.com/2226-4787/9/1/51/s1, Figure S1: Mean Likert scale scores for evaluation questionnaires for the workshop (*n* = 57). The evaluation questionnaire used a 5-point Likert scale for the first five parts, consisting of 30 items, with anchors “low” to “very high”. Questions from the evaluation questionnaire are found in the Supplementary Table S1, Table S1: Workshop Evaluation Questionnaire. Participants were asked to agree or disagree (on a scale of 1 to 5) with statements relating to the training workshop. Note, the original questionnaire and responses were in French and have been translated to English.
