# Supplementary material for: Helping Québec Pharmacists Seize the Vaccination Service Opportunity: The Pharmacy Best Practice Workshops
Source: Pharmacy (Basel). 2021 Mar 3;9(1):51. doi: 10.3390/pharmacy9010051 (PMC8005967; doi:10.3390/pharmacy9010051)

Supplementary Files To:

Helping Québec Pharmacists Seize the Vaccination Service Opportunity:

The Pharmacy Best Practice Workshops

Kajan Srirangan, PhD, MBA [1*†] and Arnaud Lavenue, PharmD, MSc[1*†]

[1]Toc Toc Communications, 104-7030 Rue Marconi, Montréal, Québec, Canada H2S 3K1

*Denotes equal contribution.

†Address correspondence to:

104-7030 Rue Marconi, Montréal, Québec, Canada H2S 3K1

Email: alavenue@toctoccommunications.com

Email: ksrirangan@toctoccommunications.com

March 3, 2021

Supplementary Table 1:

Workshop Evaluation Questionnaire. Participants were asked to agree or disagree (on a scale of 1 to 5) with statements relating to the training workshop. Note, the original questionnaire and responses were in French and have been translated to English.

Supplementary Figure 1:

Mean Likert scale scores for evaluation questionnaires for the workshop (n=57). The evaluation questionnaire used a 5-point Likert scale for the first five parts, consisting of 30 items, with anchors 'low' to 'very high'. Questions from the evaluation questionnaire are found in the **Supplementary Table 1.**

Supplementary Table 1

Workshop Evaluation Questionnaire

1- Very low (VL) **2-** Low (L) **3-** Medium (M) **4-** High (H) **5-** Very high (VH)

**PROFESSIONAL DEVELOPMENT**	**1 VL**	**2 L**	**3 M**	**4 H**	**5 VH**	**N/A**
1. This workshop will have a direct influence on my practice.	○	○	○	○	○	○
2. This workshop has allowed me to improve my level of knowledge and skills.	○	○	○	○	○	○
3. I will recommend this workshop to my co-workers.	○	○	○	○	○	○
**WORKSHOP OBJECTIVES AND CONTENT**	**1 VL**	**2 L**	**3 M**	**4 H**	**5 VH**	**N/A**
4. The learning objectives were clearly stated.	○	○	○	○	○	○
5. The content met the learning objectives.	○	○	○	○	○	○
6. The content was sufficiently detailed and precise.	○	○	○	○	○	○
7. The content was structured and easy to understand.	○	○	○	○	○	○
8. The content was organized coherently.	○	○	○	○	○	○
**KNOWLEDGE TRANSFER AND INTEGRATION**	**1 VL**	**2 L**	**3 M**	**4 H**	**5 VH**	**N/A**
9. The scenarios, clinical case studies or training questions were clearly stated.	○	○	○	○	○	○
10. The clinical case studies or scenarios were relevant and applicable to pharmacies daily.	○	○	○	○	○	○
11. The clinical case studies or scenarios were sufficient.	○	○	○	○	○	○
12. The clinical case studies or scenarios were diverse.	○	○	○	○	○	○
13. The feedback offered from the case studies or training questions allowed for knowledge transfer.	○	○	○	○	○	○
**THE FACILITATOR / SPEAKER**	**1 VL**	**2 L**	**3 M**	**4 H**	**5 VH**	**N/A**

14. The facilitator adequately presented the content and piqued my interest.	○	○	○	○	○	○
15. The facilitator had good knowledge of the subject matter.	○	○	○	○	○	○
16. The facilitator fostered interaction well.	○	○	○	○	○	○
17. The feedback offered by the speaker was relevant.	○	○	○	○	○	○
18. The speaker stated their current and potential conflicts of interest related to the workshop.	○	○	○	○	○	○
**THE ASSESSMENT**	**1 VL**	**2 L**	**3 M**	**4 H**	**5 VH**	**N/A**
19. The assessment questions reflected the content covered in the workshop.	○	○	○	○	○	○
20. The assessment measured the achievement of the learning objectives well.	○	○	○	○	○	○
21. The difficulty level of the assessment was adequate.	○	○	○	○	○	○
22. The feedback provided from the assessment was sufficient.	○	○	○	○	○	○
**GRAPHIC DESIGN AND MEDIA**	**1 VL**	**2 L**	**3 M**	**4 H**	**5 VH**	**N/A**
23. The graphics used in the teaching material were consistent and professional.	○	○	○	○	○	○
24. The graphical elements (photos, illustrations, diagrams, animations, etc.) were clear.	○	○	○	○	○	○
25. The graphical elements added value to the teaching material.	○	○	○	○	○	○
26. The downloadable documents (tools, tables, appendices) were relevant and met my needs.	○	○	○	○	○	○
27. The navigation was easy.	○	○	○	○	○	○
28. The interactive elements were working properly.	○	○	○	○	○	○
29. Case studies were well integrated into online components of the workshop.	○	○	○	○	○	○
**GENERAL STATEMENTS**	**1 VL**	**2 L**	**3 M**	**4 H**	**5 VH**	**N/A**
30. The length of the workshop activity was enough to cover the topic addressed.	○	○	○	○	○	○

31. The workshop had no commercial bias.	○ Yes<br>○ No, justify your answer
32. The online registration process was satisfactory.	○ Yes<br>○ No, justify your answer
33. How did you find out about this activity?	○ College Website<br>○ College Publications<br>○ Catalogue of the Training Portal<br>○ Employer/Colleague<br>○ Other, specify<br>______________________________
**DEVELOPMENT QUESTIONS**	

34. What were the strengths of the workshop?	
35. What improvements would you make to this workshop?	
36. What topics would you like to see addressed in future workshops?	

Supplementary Figure 1

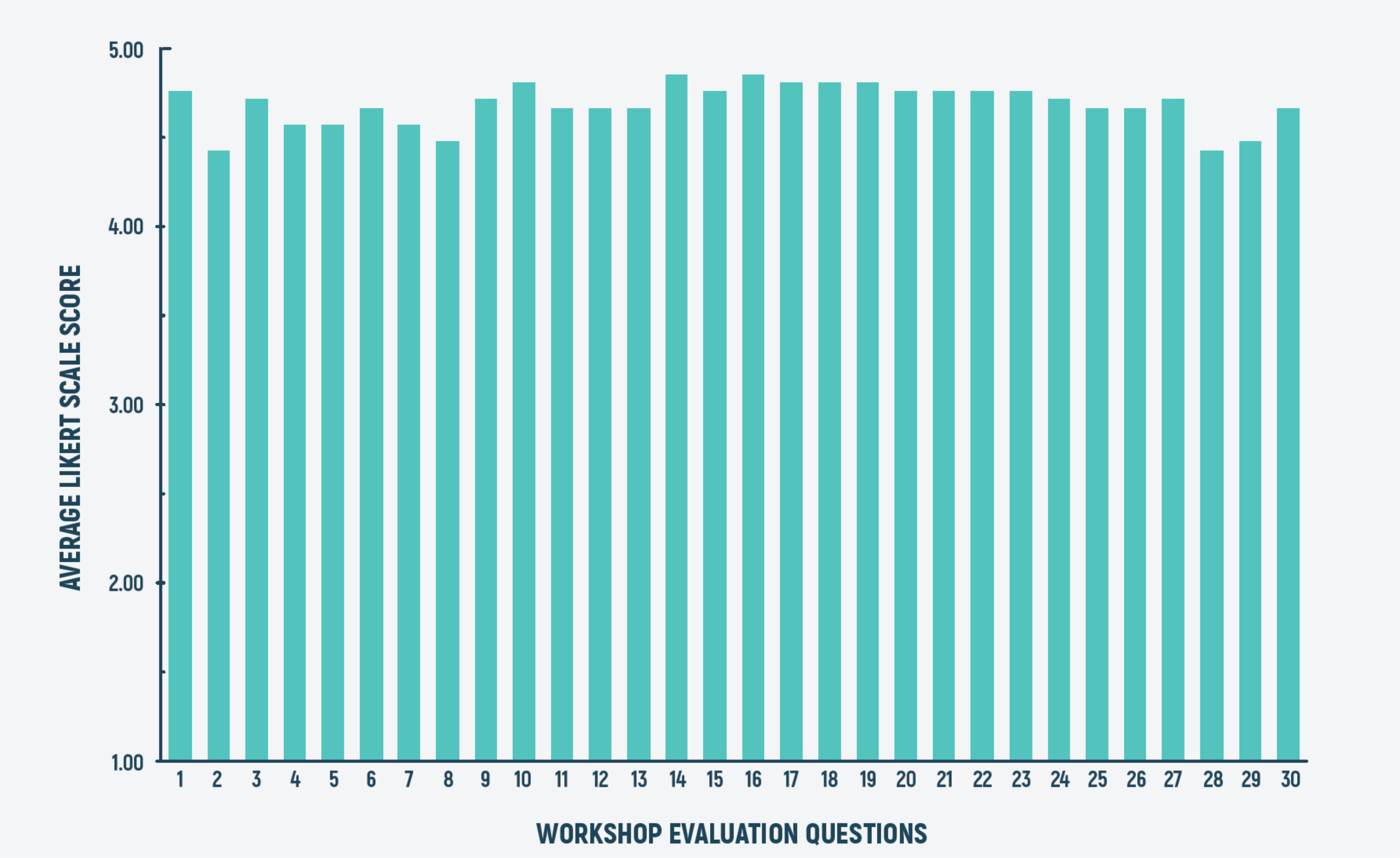

Supplement: Supplementary file 1 [file pharmacy-09-00051-s001.pdf]
